# OrthoGNC: A Software for Accurate Identification of Orthologs Based on Gene Neighborhood Conservation

**DOI:** 10.1016/j.gpb.2017.07.002

**Published:** 2017-11-11

**Authors:** Soheil Jahangiri-Tazehkand, Limsoon Wong, Changiz Eslahchi

**Affiliations:** 1 Department of Computer Science, Shahid Beheshti University, Tehran 1983969411, Iran; 2 School of Computing, National University of Singapore, Singapore 117417, Singapore

**Keywords:** Orthology, Homology, Gene neighborhood conservation, Genomic context, Comparative genomics

## Abstract

**Orthology** relations can be used to transfer annotations from one gene (or protein) to another. Hence, detecting orthology relations has become an important task in the post-genomic era. Various genomic events, such as duplication and horizontal gene transfer, can cause erroneous assignment of orthology relations. In closely-related species, gene neighborhood information can be used to resolve many ambiguities in orthology inference. Here we present OrthoGNC, a software for accurately predicting pairwise orthology relations based on **gene neighborhood conservation**. Analyses on simulated and real data reveal the high accuracy of OrthoGNC. In addition to orthology detection, OrthoGNC can be employed to investigate the conservation of **genomic context** among potential orthologs detected by other methods. OrthoGNC is freely available online at http://bs.ipm.ir/softwares/orthognc and http://tinyurl.com/orthoGNC.

## Introduction

Currently, sequencing facilities are able to produce large amounts of gene and protein sequences in a short period of time. Hence, many complete genomes of organisms are available today for more in-depth comparative studies. A first step in comparative genomics is the identification of homologous and more specifically orthologous genes. Homologous genes (homologs) are originated from a gene in the last common ancestor. In 1970, Fitch classified homologs into orthologous and paralogous genes [1]. Orthologous genes (orthologs) are homologs that have evolved by speciation event in their last common ancestor. In contrast, paralogous genes (paralogs) are homologs that have evolved by gene duplication in their last common ancestor.

Identification of orthologs is more important and of great interest, since orthologs typically tend to share a similar function [2]. Thus orthology relations can be used to transfer functional annotations (including protein–protein interactions) to newly-sequenced genomes [3], [4]. Moreover, by definition, only phylogeny of orthologs can reflect the true evolutionary history of the corresponding species correctly [5]. Therefore, only orthologs can be used to infer species phylogenies [6].

Despite the straightforward definition of orthology, the problem of assigning orthology is not trivial. Evolutionary events such as horizontal gene transfer (HGT) and gene loss often complicate the evolutionary history of genes. Hence, many methods and databases have been introduced to tackle the problem of orthology assignment. More than 40 methods and databases are listed in the “Quest for Orthologs” website (http://questfororthologs.org/orthology_databases), which can mostly be classified into two major classes according to the approaches employed.

Methods such as OrthoStrapper [7], HOGENOM [8], LOFT [9], PhylomeDB [10], and OrthoReD [11] are based on phylogenetic analysis. Phylogeny-based methods seem to be more precise, with high specificity reported [7], [12], [13]. However, ambiguities in the inferred gene trees and species trees, as well as wrong placement of the root can lead to incorrect assignment of orthologs. In addition, these methods require large computational cost, making their usage impractical for large datasets [14], [15], [16].

**Figure 1:**
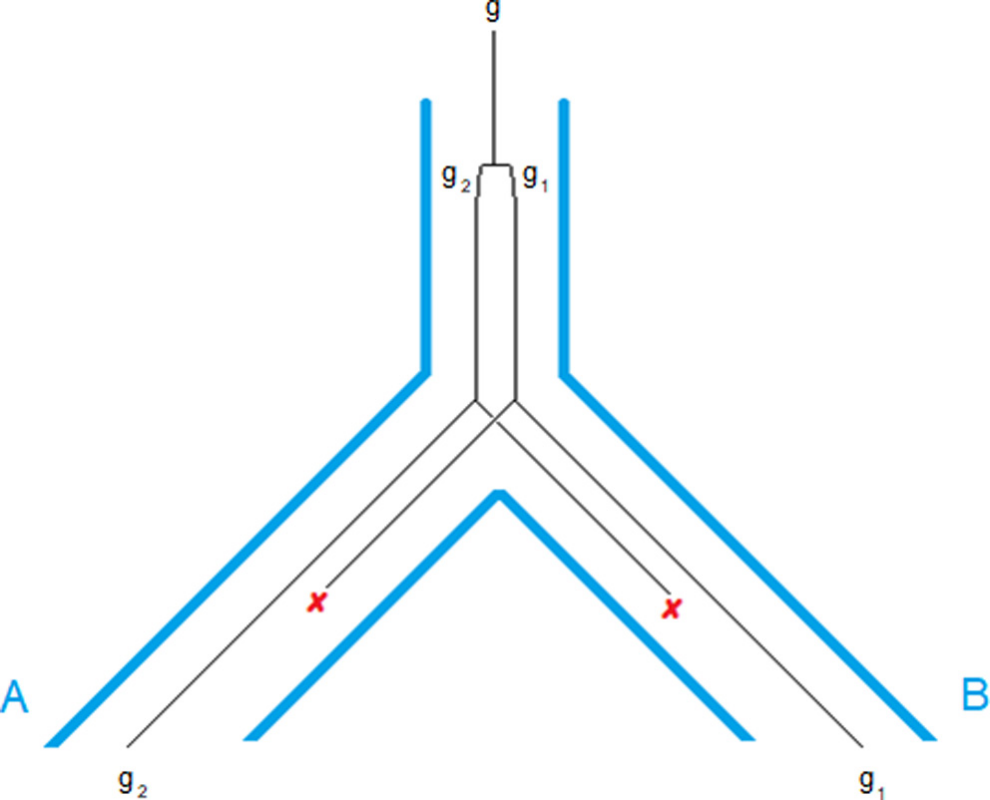
**An example for lineage-specific gene loss** Suppose gene g has undergone a duplication event, resulting in two genes, g_1_ and g_2,_ which is followed by a speciation event. Deletion of g_1_ in lineage A and deletion of g_2_ in lineage B can lead to the wrong assignment of g_2_ from lineage A and g_1_ from lineage B as orthologs. According to the duplication event in the last common ancestor, g_1_ and g_2_ are paralogs. Since duplication event occurs prior to speciation, g_1_ and g_2_ are out-paralogs.

The second class of methods usually employs a clustering algorithm on a weighted graph that is built from pairwise sequence similarities. Examples in this class include OMA [17], OrthoMCL [18], InParanoid [19], Proteinortho [20], and OrthoDB [21]. These methods, because of their tractability, have gained more popularity, particularly when used for large datasets. However, these methods are based on the molecular clock hypothesis by assuming that orthologous sequences are more similar and would fail to detect orthologs when the molecular clock hypothesis is violated [22]. Furthermore, HGT and convergent evolution as well as linage-specific gene loss (Figure 1) can introduce false positive relations. Note that duplications prior to a speciation event (in-paralogs) and duplications after a speciation event (out-paralogs) [23] introduce one-to-many or many-to-many orthology relation, further complicating the process of orthology detection. Most similarity-based methods that employ clustering, present the orthology relations as ortholog groups instead of pairwise relations. As a result, these groups can contain in-paralogs and out-paralogs, making their usage inappropriate for studies such as phylogeny inference where one-to-one orthology relations are needed.

To increase the quality of inferred orthologs, some methods attempt to take genomic context into account [24], [25], [26], [27], [28], [29]. Due to genome rearrangements, as well as gene gains/losses, genomic context is less conserved beyond the genus level. Nonetheless, it can be a strong evidence of orthology when found. Conservation of gene neighborhood can assist in distinguishing orthologs from out-paralogs [30], [31]. Similarly, it can prevent misinterpretation of HGTs and genes with convergent evolution as orthologs. Another interesting advantage of employing genomic context is to reveal orthologous genes or proteins that share low sequence similarities [32]. These orthologs can be missed in clustering or homology inference steps due to trade-off in favor of specificity. Moreover, with the advent of next-generation sequencing, many closely related genomes are available today. As a result, comparative studies are extended to closely related species and even strains of a same species where genomic context is highly conserved.

In this paper, we extend the method of Jun et al. [29] in both homology detection and orthology detection to increase the accuracy of predictions. We propose OrthoGNC, a similarity-based method and a software that outputs high-quality pairwise orthology relations based on gene neighborhood conservation. Moreover, OrthoGNC can be employed to investigate the conservation of genomic context among potential orthologs detected by other methods.

## Method

Jun et al. tested a simple method based on gene neighborhood conservation to extract orthology relations in mammalian proteomes [29]. According to their method, two genes are orthologous if they are homologous and share at least one homologous neighbor in a neighborhood size of three upstream and three downstream genes. Also, homology between two genes are defined as Blastp E-value < 1e−5. We have extended this method in both homology detection and orthology detection. Similar to the aforementioned method, OrthoGNC performs three main steps to infer orthology relations: (i) computing pairwise sequence similarities, (ii) identifying homologous sequences, and (iii) inferring orthologs according to gene neighborhood conservation. However, in each step, parameters can be set to various values to provide desired output. These parameters can be easily adjusted by the user in a configuration file or using a user-friendly GUI. Moreover, in OrthoGNC, inference of orthology relation can be done in an iterative routine to produce more accurate and sensitive results.

### OrthoGNC steps

OrthoGNC is implemented in Java and accepts both DNA and protein sequences in FASTA format. In addition, the order of appearance of genes or proteins in the FASTA file must be the same as the order of their appearance in the underlying genomes.

#### Step 1

Like other similarity-based methods, OrthoGNC requires pairwise sequence similarities at the very beginning step. The well-known and widely-used heuristic software BLAST is used to compute the similarity score [33]. OrthoGNC can run BLAST in multiple threads in parallel, thus improving the running time of the BLAST step significantly in multi-core CPUs. The number of simultaneous BLAST jobs can be set or auto detected according to the number of available cores and the input parameters of BLAST, such as E-value and substitution matrix, can be adjusted by the user.

#### Step 2

In the second step, OrthoGNC infers homology relations using pairwise similarity scores. Two sequences are assumed to be homologous if they share a significant sequence similarity (30%–35% for proteins as a rule of thumb according to Ref. [14]). To infer homology relation, OrthoGNC looks into BLAST hits to make sure that not only a certain amount of identical residues is matched but also a certain length of both sequences is covered. The minimum percentage of identical residues (*T*_i_) and minimum percentage of coverage (*T*_c_) can be adjusted by the user.

#### Step 3

In the third and last step, OrthoGNC extracts orthology relations from homologous sequences based on gene neighborhood conservation. Similar to the adaptive RBAH [20], OrthoGNC uses a ratio *T*_b_, 0 < *T*_b_ < 1, to tolerate possible variances of molecular clock rate. This allows every homolog of a gene that has a score > *T*_b_ *× score of the best homolog* to be an orthology candidate for that gene. Afterward, genes and their candidate orthologs are investigated to determine the number of common homologous neighbors, which can be done via one of two predefined routines, namely One2One mapping and unique intersection. In the first routine, each of the 2n neighbors of a gene (n upstream and n downstream) is checked against its corresponding gene in the neighborhood of candidate orthologs to see whether they are homologs (Figure S1A). If the number of homologous pairs exceeds a predefined threshold, then the gene and its candidate ortholog are *bona fide* orthologs. In the second routine, OrthoGNC counts the unique homologous matches between neighbors of a gene and neighbors of its candidate ortholog without considering co-linearity (Figure S1B). Similar to the first routine, if the number of unique homologous matches exceeds a predefined threshold then the gene and its candidate ortholog are *bona fide* orthologs. Consideration of gene order in the first routine makes it more stringent while, in contrast, the second routine allows for local rearrangement and gene gains/losses. We also observed that other orthology detection parameters – maximum tolerance ratio (*T*_b_), radius of neighborhood (*N*), and minimum number of common neighbors (*T*_n_) – can also affect the number of inferred orthologs dramatically. By relaxing these parameters, OrthoGNC is able to find more orthologs; however, more false-positive relations would also be introduced. To maintain the precision of inferred orthologs, OrthoGNC identifies orthology relations iteratively, that is, the user can define more than one parameter set for multiple rounds of orthology inference. Accordingly, if in certain round of orthology inference with certain set of parameters, OrthoGNC finds an ortholog for a gene in a strain, it does not look for another ortholog of this gene in the same strain in the subsequent rounds. To clarify, suppose we find the orthology relation (g, x) in some round; in the next iterations with more relaxed parameters, new homology relations such as (g, y) and (h, x) might be introduced where y and h are in the same genomes as g and x, respectively. Inference of (g, x) using more stringent parameters implies that (g, y) and (h, x) are wrong unless (g, h) and (x, y) duplicated after the speciation event.

The number of orthology-detection rounds and the parameters in each round – *T*_b_, *N*, *T*_n_, and the neighbor investigation routine (NIR) to be used – can be easily configured by the user. Finally, the pairwise orthology relations are reported for each pair of input genomes.

Although the main objective of OrthoGNC is to deliver highly accurate and precise orthology relations, it can be combined with other methods to achieve higher recall. To this end, user can choose to combine the output of OrthoGNC with an arbitrary set of orthologs that is predicted by another method. In this case, if OrthoGNC is unable to find any ortholog for gene g from strain S, it outputs genes from S that are introduced as orthologs of g by the other method.

### Benchmarking

Evaluation of orthology inference methods is not an easy task, because we do not know the true evolutionary history of genes. Recently, in an effort to standardize orthology benchmark [34], a public web service has been introduced to assess different methods on 66 species. Unfortunately, all of these species are evolutionary distant (beyond the genus level), making it inappropriate for our study. We thus compared OrthoGNC to other methods on both simulated and real data, notwithstanding that high conservation of genomic context is a strong and self-verifying criterion in orthology inference and has already been evaluated [29], [35]. Four similarity-based methods that produce pairwise orthology relations are selected for comparison, including OMA [17], InParanoid [19], Proteinortho [20], and EGM2 [36]. In addition, OrthoGNC is also compared to Jun et al.’s method (Figure S2), which is now a special case of OrthoGNC, where E-value = 1e−5, *T*_i_ = 0%, *T*_c_ = 0%, *N* = 3, *T*_n_ = 1, *T*_b_ = 0.0, and NIR = “unique intersection”.

OMA, InParanoid, and Proteinortho all use clustering for orthology inference, while EGM2 employs genomic context to perform iterative graph matching. The latest version of each software was acquired from their official website, and was run with default parameters. For OrthoGNC, we used different parameter configurations (Table 1) to evaluate the effect of parameters chosen on ortholog inference in practice. We first used each configuration (Conf) to infer the orthologs in single rounds. Then, we used configurations 2–8 in sequence to infer the orthologs iteratively, with homology detection parameters fixed for all iterative rounds. We further show how OrthoGNC could be employed to refine the orthology relations that are predicted by other methods. Different parameter configurations (Table S1) have also been done to assess the impact of gene neighborhood conservation (Figure S3).

**Table 1:** **Parameter configurations used for performance evaluation of OrthoGNC**

**Parameter**		**Conf 1**	**Conf 2**	**Conf 3**	**Conf 4**	**Conf 5**	**Conf 6**	**Conf 7**	**Conf 8**
Homology	E-value	10^−5^	10^−5^	10^−5^	10^−5^	10^−5^	10^−5^	10^−5^	10^−5^
	*T*_i_	30%	0%	0%	0%	0%	0%	0%	0%
	*T*_c_	50%	0%	0%	0%	0%	0%	0%	0%

Orthology	*N*	7	7	7	7	7	7	7	0
	*T*_n_	9	9	9	7	5	3	1	0
	*T*_b_	0.80	0.80	0.80	0.80	0.80	0.80	0.80	1.0
	NIR	O	O	I	I	I	I	I	I

*Note*: *T*_i_, minimum percentage of identical matches in a BLAST hit; *T*_c,_ minimum percentage of coverage of query and subject sequences in BLAST hit; *N*, radius of neighborhood to be investigated; *T*_n_, minimum number of common neighbors (0 ≤ *T*_n_ ≤ 2 * *N*); *T*_b_, maximum tolerance ratio from score of best hit (0 ≤ *T*_b_ ≤ 1); NIR, neighborhood investigation routine. I stands for unique intersection and O stands for One2One mapping.

For performance evaluation, we applied all methods on two datasets; a simulated proteome dataset and a prokaryotic proteome dataset. Prokaryotic genomes are known to be fluid [37], and many genes are subject to lose their ancestral order, due to significant amounts of rearrangements. We show that even in the presence of many rearrangements, genomic context is still highly informative in detecting accurate orthology relations for closely-related species.

#### Simulated data

In the absence of a gold standard, we have used the Artificial Life Framework (ALF) [38] to simulate a proteome set consisting of 30 species. ALF was previously employed for simulating bacteria-like and mammalia-like genomes to assess the impact of different evolutionary forces on orthology inference [39]. We used the same set of parameters that was used to generate bacteria-like genomes in an earlier work, by incorporating genome rearrangement event in addition to other predefined evolutionary events.

#### Real data

The real dataset comprises eight proteomes from genus *Mycobacterium*. The included species are *Mycobacterium ulcerans* Agy99 (4241 proteins), *Mycobacterium leprae* TN (1605 proteins), *Mycobacterium avium* subsp. paratuberculosis K-10 (4350 proteins), *Mycobacterium smegmatis* str. MC2 155 (6716 proteins), *Mycobacterium bovis* AF2122/97 (3920 proteins), *Mycobacterium marinum* M (5452 proteins), *Mycobacterium tuberculosis* H37Rv (3989 proteins), and *Mycobacterium abscessus* (4941 proteins). All proteomes were acquired in FASTA format from PATRIC [40].

#### Evaluation

For the simulated dataset, we first count the number of correctly-predicted orthology relations (true positive; TP), the number of incorrectly-predicted orthologs (false positive; FP), and the number of missed orthology relations (false negative; FN). We then calculate the precision and recall for each method according to Eq. (1).

(1) $$\mathrm{Precision}=\frac{\text{TP}}{\text{TP}+\text{FP}}\text{,}\;\mathrm{Recall}=\frac{\text{TP}}{\text{TP}+\text{FN}}$$

For real data, we cannot calculate precision and recall due to unknown orthology relations. Instead, we investigate how many genes for which OrthoGNC predicted an ortholog while each competing method was unable to find any orthologs or suggested another ortholog. To this end, we calculate three sets of orthology relations, namely, *M*_Method_, *M*′_Method_, and *M*″_Method_ according to (2). For instance, to obtain *M*_OMA,_ we look for every orthology relation (g, o) predicted by OrthoGNC, where OrthoGNC predicted ortholog *o* in species *S* for gene *g*, and OMA failed to predict any ortholog for *g* in *S*. For *M*′_OMA_, we look for every orthology relation (g, o) predicted by OrthoGNC, where OrthoGNC predicted ortholog *o* in species *S* for gene *g* and OMA predicted some other ortholog for *g* in *S*. For *M*″_OMA,_ we look for every orthology relation (g, o′) predicted by OMA, where OMA predicted ortholog *o*′ in species *S* for gene g and OrthoGNC predicted some other ortholog for *g* in *S*. To put it simple, for a total number of |*M*′_OMA_| genes OrthoGNC predicted orthologs while for the same genes OMA predicted a total number of |*M*″_OMA_| other orthologs. Furthermore, we calculate the set U_OrthoGNC_ of orthologous genes that were only predicted by OrthoGNC. We computed *M*_Method_, *M*′_Method_, *M*″_Method_, and *U*_OrthoGNC_ for both the simulated data and real data

(2) $$M_{\text{Method}}={⋃}_{S\in \text{Species}}\{(g.o)|o\in S\wedge (g.o)\in \text{OrthoGNC}\wedge ∄o^{\prime }\in S:(g.o^{\prime })\in \text{Method}\}M_{\text{Method}}^{\prime }={⋃}_{S\in \text{Species}}\{(g.o)|o\in S\wedge (g.o)\in (\text{OrthoGNC}-\text{Method})\wedge (\exists o^{\prime }\in S:o\neq o^{\prime }\wedge (g.o^{\prime })\in (\text{Method}-\text{OrthoGNC}))\}M_{\text{Method}}^{\prime \prime }={⋃}_{S\in \text{Species}}\{(g.o^{\prime })|o^{\prime }\in S\wedge (g.o^{\prime })\in (\text{Method}-\text{OrthoGNC})\wedge (\exists o\in S:o\neq o^{\prime }\wedge (g.o)\in (\text{OrthoGNC}-\text{Method}))\}U_{\text{OrthoGNC}}=\text{OrthoGNC}-(\text{OMA}\cup \text{ProteinOrtho}\cup \text{Inparanoid})$$

For real data, in addition to calculating *M*_Method_, *M*′_Method_, *M*″_Method_, and *U*_OrthoGNC_, we built Venn diagrams using the online tool InteractiVenn [41] to provide an overall picture of the predicted orthologs for all methods. Pairwise orthology relations are introduced to InteractiVenn as a string value, in which two protein ids are separated with a delimiter character. For all predicted x–y orthology relations, we also add the y–x relations manually.

## Results and discussion

In order to evaluate OrthoGNC and the effect of parameters chosen on predicting orthologs, we ran OrthoGNC using different configurations of parameters both in single rounds and iteratively (Table 1). In single rounds, we only used one parameter configuration for orthology inference, whereas in iterative mode, rounds of orthology inference were performed with parameters of Conf 2–8 sequentially. For example, in order to iteratively infer orthologs with Conf 4, three rounds of orthology inference with Conf 2–4 is done. If in a round of orthology inference, OrthoGNC finds an ortholog for gene *g* in a strain, it does not look for another ortholog of gene *g* in the same strain in the subsequent rounds.

### Ortholog inferring performance of OrthoGNC on simulated data

**Figure 2:**
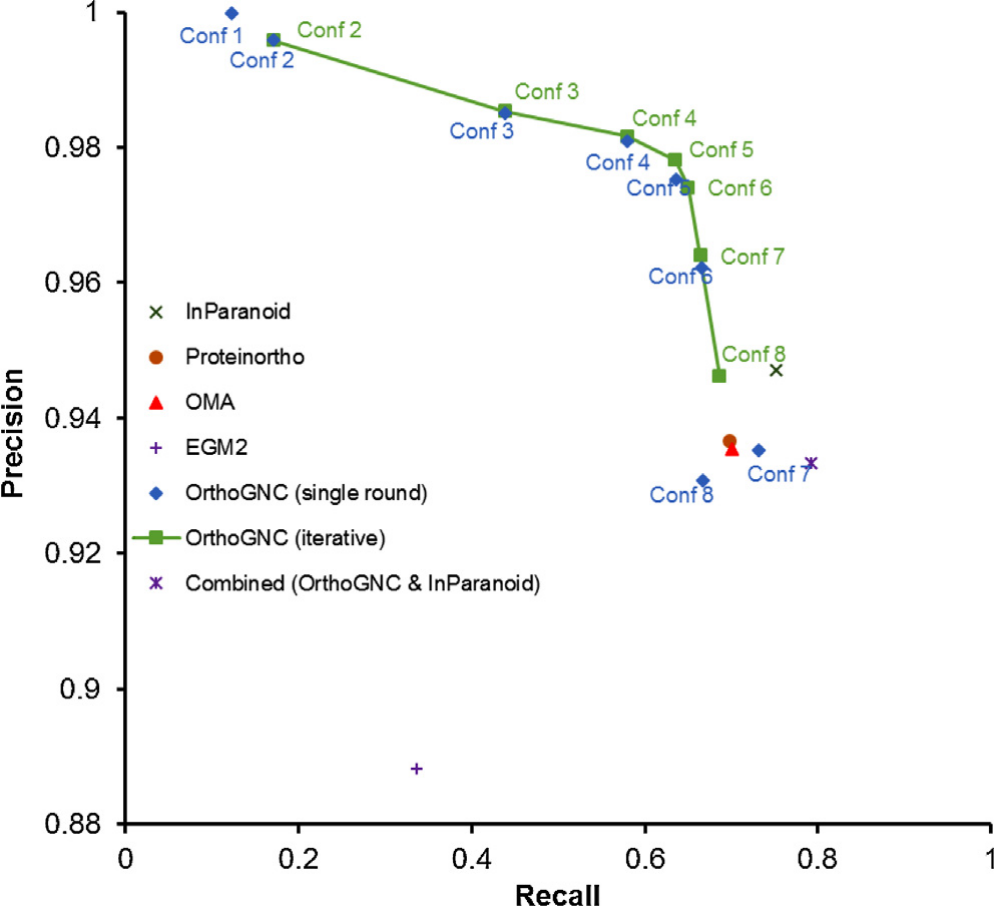
**Precision–recall plot of OrthoGNC and other methods on simulated data** Precision and recall rates for OrthoGNC in single rounds and iteratively are compared with those using Proteinortho, OMA, InParanoid, EGM2, as well as combination of OrthoGNC and InParanoid. Parameter configurations for Conf 1–8 are listed in Table 1. Conf, configuration.

The ortholog inferring performance on the simulated data using various methods is shown in Figure 2. Precision of OrthoGNC converges to one by choosing strict parameters as in Conf 1. However, the amount of predicted orthology relations decreases when parameters are set strictly to output stringent results. It is of note that, even in this case, OrthoGNC is able to find orthology relations that may not be found by any other method. As seen in Table 2 (*M*_Method_), with the stringent parameter set of Conf 1, 2670 (and 99.96% of these are correct), 3750 (99.89%), 1648 (100%), and 37,893 (99.97%) orthology relations predicted by OrthoGNC were missed by Proteinortho, OMA, InParanoid, and EGM2, respectively. Moreover, with Conf 1, 332 (100% of these are correct) orthology relations (= |*U*_OrthoGNC_|) found by OrthoGNC are missed by all other methods. By relaxing homology inference parameters in Conf 2, OrthoGNC detects many more new orthology relations that are missed by other methods, although a small amount of false positives is included. This is because other methods use higher similarity cutoffs to maintain the precision, while in OrthoGNC, in presence of its gene neighborhood conservation criteria, the sequence similarity parameters can be relaxed to include more distant orthologs.

**Table 2:** **Orthology relations obtained on simulated data**

**Method**	**Parameter**	**Conf 1 (TP %)**	**Conf 2 (TP %)**	**Conf 3 (TP %)**	**Conf 4 (TP %)**	**Conf 5 (TP %)**	**Conf 6 (TP %)**	**Conf 7 (TP %)**	**Conf 8 (TP %)**
Proteinortho	*|M|*	2670 (99.96%)	8035 (94.47%)	26,628 (86.46%)	40,244 (84.59%)	46,842 (82.73%)	49,337 (81.73%)	53,193 (79.87%)	59,791 (75.50%)
	*|M*′*|*	778 (99.87%)	1455 (96.28%)	4773 (88.85%)	7165 (88.62%)	8312 (88.05%)	8715 (87.32%)	9363 (84.97%)	9678 (82.55%)
	*|M*″*|*	831 (62.57%)	1542 (68.54%)	5027 (68.27%)	7563 (66.49%)	8776 (65.92%)	9220 (65.77%)	9857 (65.70%)	10,175 (66.37%)

OMA	*|M|*	3750 (99.89%)	8508 (93.88%)	31,587 (85.75%)	47,741 (84.11%)	55,802 (82.83%)	58,244 (81.93%)	62,645 (80.15%)	71,587 (76.39%)
	*|M*′*|*	420 (100.0%)	700 (97.42%)	2401 (90.17%)	3681 (89.78%)	4294 (89.33%)	4477 (88.29%)	4937 (84.44%)	5239 (80.37%)
	*|M″|*	443 (49.43%)	751 (52.86%)	2586 (51.81%)	3974 (50.42%)	4652 (49.39%)	4864 (49.60%)	5358 (50.83%)	5677 (52.61%)

InParanoid	*|M|*	1648 (100.0%)	3590 (87.35%)	13,082 (72.80%)	20,201 (70.05%)	24,211 (67.86%)	25,527 (67.07%)	27,674 (65.57%)	32,215 (60.69%)
	*|M*′*|*	936 (100.0%)	1552 (95.10%)	4752 (84.15%)	7167 (83.49%)	8368 (83.23%)	8756 (82.60%)	9456 (79.52%)	9979 (75.71%)
	*|M*″*|*	989 (45.29%)	1660 (50.30%)	5087 (50.52%)	7693 (49.17%)	8964 (48.12%)	9377 (47.97%)	10,062 (49.09%)	10,612 (51.03%)

EGM2	*|M|*	37,893 (99.97%)	62,145 (99.12%)	186,004 (97.41%)	257,415 (96.89%)	287,085 (96.40%)	296,488 (95.89%)	307,842 (94.76%)	327,602 (92.52%)
	*|M*′*|*	179 (100.0%)	327 (99.38%)	979 (90.70%)	1412 (88.59%)	1646 (88.57%)	1717 (87.07%)	1794 (85.67%)	1861 (83.28%)
	*|M*″*|*	226 (39.82%)	393 (42.23%)	1068 (48.87%)	1529 (50.03%)	1788 (50.50%)	1869 (49.97%)	1947 (49.82%)	2014 (49.15%)
	*|U*_OrthoGNC_*|*	332 (100.0%)	1744 (71.90%)	8694 (48.26%)	14,262 (46.66%)	17,464 (45.21%)	18,456 (44.53%)	20,160 (43.51%)	24,836 (40.42%)

*Note*: Different homology inference parameters are used for Conf 1 (single round), while Conf 2 (single round) is the starting configuration for iterative rounds. For Conf 3–8, only results for iterative rounds are shown here due to the high accuracy obtained by iterative orthology inference. *U*_OrthoGNC_ represents orthologous genes only predicted by OrthoGNC. For each competing method, M represents orthology relation (g, o) predicted by OrthoGNC, where OrthoGNC predicted ortholog *o* in species *S* for gene *g*, but the competing method failed to predict any ortholog for *g* in *S*. *M*′ represents orthology relations (g, o) predicted by OrthoGNC, where OrthoGNC predicted ortholog *o* in species *S* for gene *g*, but the competing method predicted some other ortholog for *g* in *S*. *M*″ represents orthology relation (g, o′) predicted by a competing method, where the method predicted ortholog *o*′ in species *S* for gene *g*, but OrthoGNC predicted some other ortholog for *g* in *S*. Conf, configuration; TP, true positive.

Another advantage of OrthoGNC is to distinguish the main ortholog [35] in the presence of multiple candidates. Accordingly, we were curious about the number of genes for which an ortholog with conserved neighborhood exist and are found by OrthoGNC while other methods predicted other orthologs. To this end, we calculated the sets *M*′_Method_ and *M*″_Method_.

As depicted in Table 2, with Conf 1, there are 936 orthology relations (g, g′) in set *M*′_InParanoid_, such that OrthoGNC correctly predicted gene *g*′ from species *S* as an ortholog for *g*, while InParanoid predicted some other ortholog(s) like *o* from *S* as an ortholog for *g*, resulting in 989 (co-)orthology relations like (g, o) in *M*″_InParanoid_. Within these 989 (co-)orthology relations in *M*″_InParanoid_ that are not identified by OrthoGNC, only 45.29% are true positives. |*M*′_Method_| and |*M*″_Method_| are slightly better for Proteinortho, OMA, and EGM2 (Table 2). Again, note the increase in |*M*′_Method_| and |*M*″_Method_| when parameters of OrthoGNC are relaxed.

Also, it is shown that iterative inference of orthology relations can slightly improve the precision (green square in Figure 2). By relaxing parameters in subsequent rounds, OrthoGNC achieves higher recall while preserving the precision. This is more interesting for Conf 8, where the neighborhood conservation criteria were completely relaxed. In fact, it only detects orthologous genes that are reciprocally best hit (RBH). In single-round inference, RBH algorithm misses some of the orthologous genes that deviated from the molecular clock assumption. Moreover, not all RBHs are necessarily orthologous [22]. With iterative inference, these relations are dismissed if a relation satisfying the neighborhood conservation criterion exists in previous rounds. Corresponding values of |*M*_Method_|, |*M*′_Method_|, and |*M*″_Method_| for iterative orthology inference with Conf 2–8 are shown in Table 2.

Another interesting observation is that *M*_Method_ and *M*′_Method_ have consistently higher true-positive rate than *M*″_Method_ (Table 2). This suggests that when OrthoGNC disagrees with another method on the orthologs of a gene, orthologs reported by OrthoGNC are generally more accurate than those reported, if any, by the other method.

The main objective of OrthoGNC is to deliver highly sensitive and precise orthology relations. However, as shown in Figure 2, OrthoGNC (Conf 7 single round) is superior to both OMA and Proteinortho in recall at almost the same level of precision. However, one might argue that the lower recall of OrthoGNC than InParanoid may result in a smaller F-measure. To achieve a higher recall, the user can choose to combine the output of OrthoGNC with any set of orthologs that is inferred by other methods as described in Methods. For instance, combination of OrthoGNC (Conf 7 iterative) with InParanoid improved recall, thus resulting in a higher F-measure (0.8570) than both OrthoGNC (0.7961) and InParanoid (0.8385) alone (Figure 2).

### Ortholog inferring performance of OrthoGNC on real data

**Figure 3:**
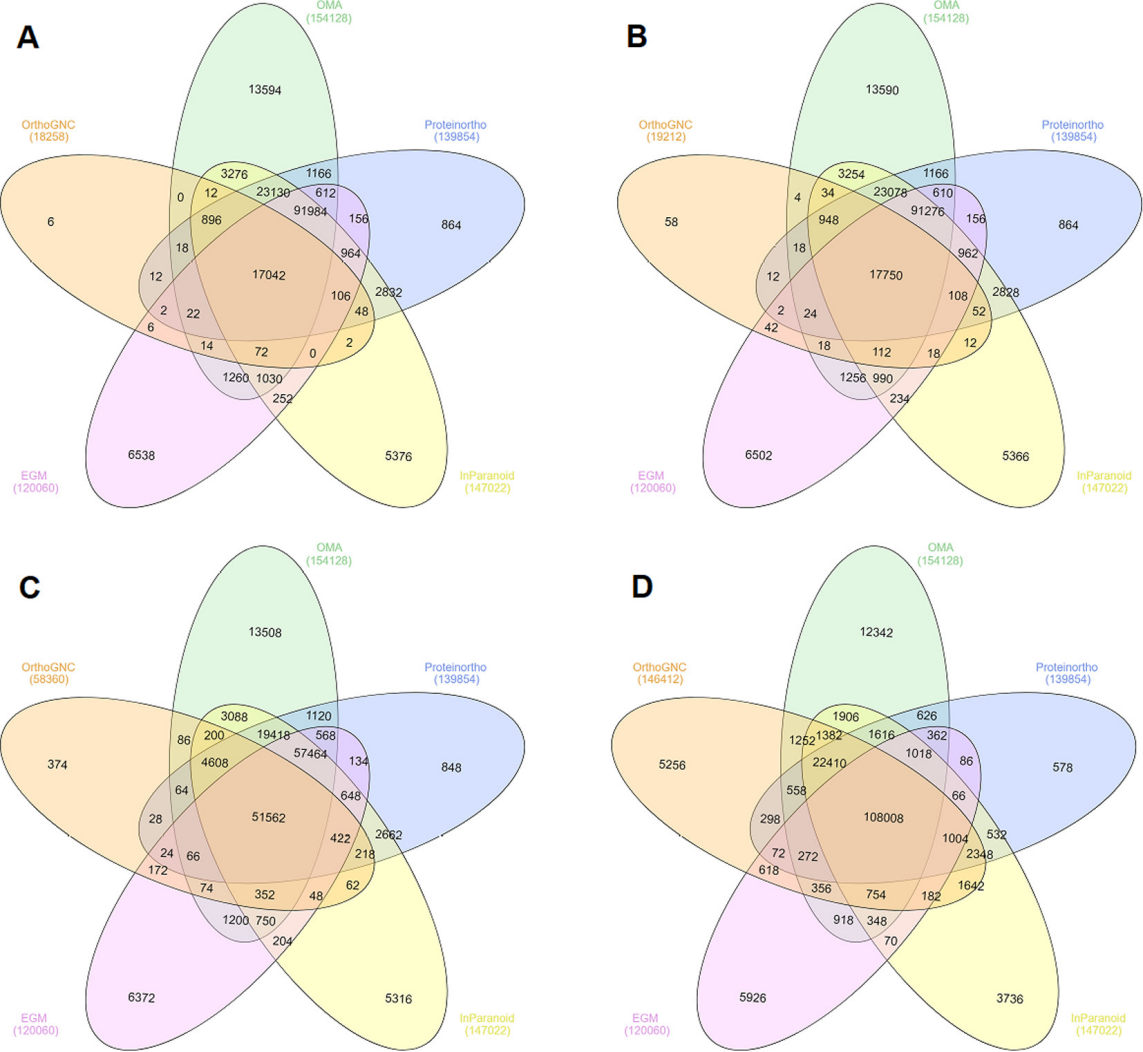
**Performance of OrthoGNC and other methods on eight *Mycobacterium* species** Orthologs predicted by OrthoGNC using Conf 1 (**A**), Conf 2 (**B**), Conf 3 (iterative; **C**) and Conf 8 (iterative; **D**) were compared with those by Proteinortho, OMA, InParanoid, and EGM2 using Venn diagrams. Parameter configurations for Conf 1, 2, 3, and 8 are listed in Table 1.

For real data, we compare the inference output of various methods using Venn diagrams. Corresponding Venn diagrams for Conf 1, 2, 3 (iterative), and 8 (iterative) are depicted in Figure 3. We also computed *M*_Method_, *M*′_Method_, *M*″_Method_, and *U*_OrthoGNC_ for this dataset (Table 3). Our results show that although prokaryotic genomes are known to be fluid, gene neighborhood is still highly informative in detecting orthologs at genus level. In particular, some orthology relations are only detected by OrthoGNC. Moreover, orthology inference parameters as stringent as Conf 1 or 2 appear not necessary. Specifically, by changing NIR to “unique intersection” in Conf 3, 39,148 more orthology relations are detected in comparison to Conf 2; out of which, 38,068 (97.24%) relations are also detected by at least three other methods (Figure 3B and C). This observation confirms the high degree of local rearrangements in prokaryotic genomes [37]. Therefore, one can relax the gene neighborhood investigation method according to the genomes studied to allow more local rearrangements. Even with iterative inference by Conf 8 (Figure 3D), 132,448 out of 146,412 (90.46%) orthology relations detected by OrthoGNC are also predicted by at least three other methods, indicating the predictions by OrthoGNC agree well with the intersection of three other methods. Therefore, in addition to inferring accurate relations based on high conservation of genomic context, OrthoGNC is also able to infer much more relations that are consistent with other state-of-art methods.

**Table 3:** **Orthology relations obtained on real data**

**Method**	**Parameter**	**Conf 1**	**Conf 2**	**Conf 3**	**Conf 4**	**Conf 5**	**Conf 6**	**Conf 7**	**Conf 8**
Proteinortho	*|M|*	101	283	1173	1872	2548	3407	7470	9160
	*|M*′*|*	11	15	95	167	313	488	1170	1268
	*|M*″*|*	11	16	103	173	329	554	1091	1190

OMA	*|M|*	174	292	1164	1804	2418	3217	6743	9262
	*|M*′*|*	8	12	108	193	318	492	1278	1474
	*|M*″*|*	8	13	136	294	488	791	2329	2589

InParanoid	*|M|*	57	153	673	1098	1574	2251	5506	6424
	*|M*′*|*	23	25	114	207	338	517	1139	1271
	*|M*″*|*	27	30	123	217	379	596	1328	1480

EGM2	*|M|*	985	1125	5553	8986	12,695	16,654	26,311	34,140
	*|M*′*|*	9	13	56	112	179	259	597	635
	*|M*″*|*	15	22	84	151	227	359	791	838
	*|U*_OrthoGNC_*|*	6	58	374	650	992	1488	4434	5256

*Note*: Different homology inference parameters are used for Conf 1 (single round), while Conf 2 (single round) is the starting configuration for iterative rounds. For Conf 3–8, only results for iterative rounds are shown here due to the high accuracy obtained by iterative orthology inference. Conf, configuration; TP, true positive.

### Orthology refinement

**Figure 4:**
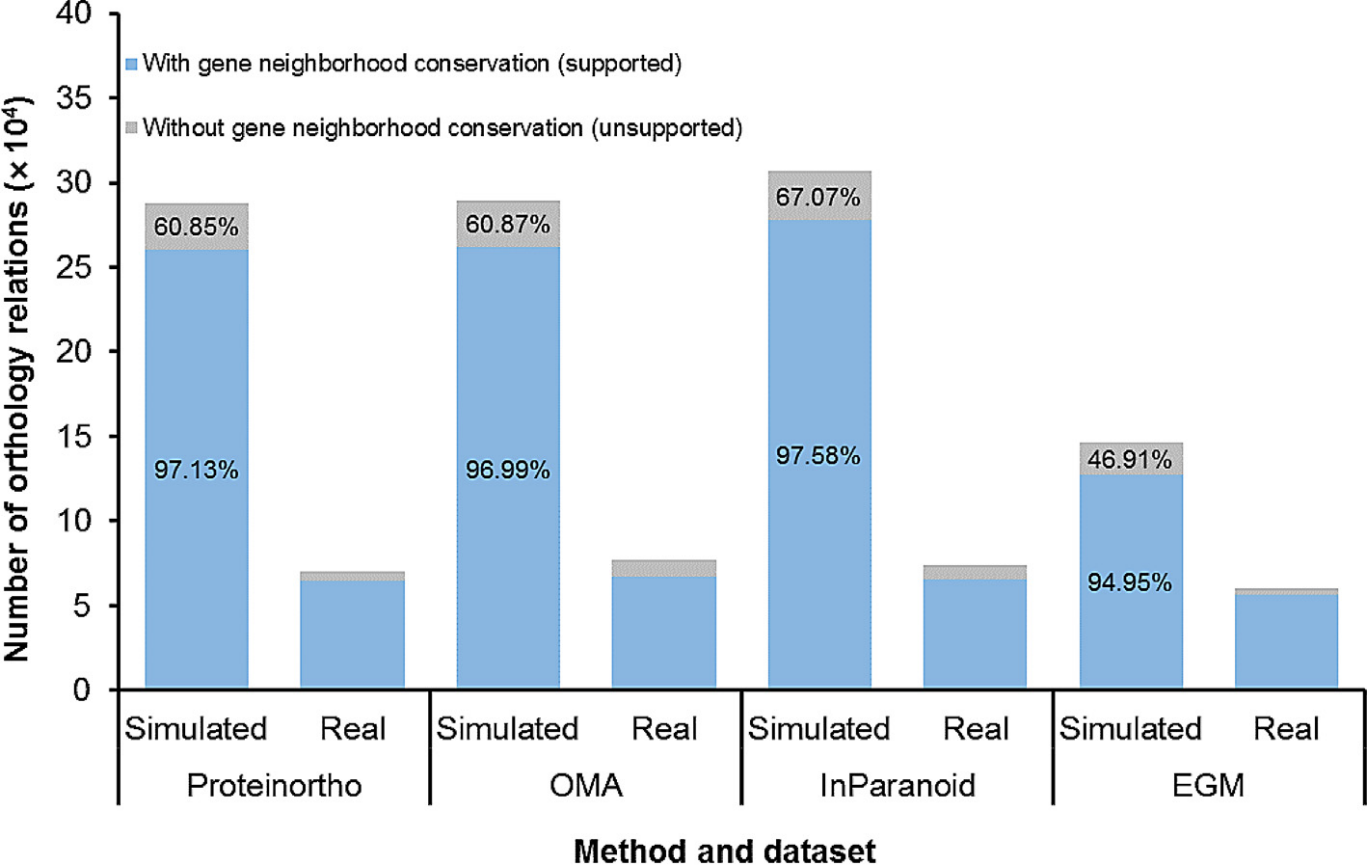
**Gene neighborhood conservation of predicted orthology using four other methods** For each method, the stacked bar on the left side indicates the performance for the simulated data and the right one for the real data. The blue bars depict the number of orthology relations that follow the predefined parameters (E-value = 1e−02, *T*_i_ = 0, *T*_c_ = 0, *N* = 7, *T*_n_ = 1, and NIR = “unique intersection”) and the gray bars indicate the number of orthology relations without gene neighborhood conservation. Precision of predicted orthologs on simulated data is provided in percentage on the bars for both supported (blue) and unsupported (gray) relations.

In addition to orthology inference, OrthoGNC has a separate interface for refining a given set of orthology relations by investigating gene neighborhood conservation among them. For the input genomes (or proteomes) and a set of input orthologs provided by the user, OrthoGNC investigates the given relations to see whether they follow a user-defined degree of gene neighborhood conservation. As a result, the input relations get processed and saved into two separate files: one for relations that are supported by gene neighborhood conservation and one for the relations without the support. We tested this feature on orthologs predicted by the other four methods on both simulated and real data, by setting easy parameters to investigate a minimal gene neighborhood conservation (E-value = 1e−02, *T*_i_ = 0, *T*_c_ = 0, *N* = 7, *T*_n_ = 1, and NIR = “unique intersection”). Percentage of correct orthologs for both supported and unsupported relations was calculated for the simulated data. As shown in Figure 4, the percentage of correctly-predicted relations are significantly higher for supported relations than unsupported ones.

### Running time comparison

We also compared the running time of OrthoGNC with other methods. To do this, we ran all methods on two proteomes (*M. ulcerans* Agy99 and *M. tuberculosis* H37Rv) using a personal computer with an Intel Core i7-4702MQ 2.20 GHz processor and 6 Giga bytes of RAM. As Proteinortho automatically sets the number of concurrent threads to available cores, we manually set the number of threads to 8 for OMA and OrthoGNC to facilitate comparison; however, InParanoid and EGM2 accept no parameter on the number of concurrent threads. As shown in Table 4, only EGM2 is faster than OrthoGNC, probably because EGM2 uses its own heuristics instead of BLAST to compute the similarities.

**Table 4:** **Running times of different methods on proteomes of *M. ulcerans* Agy99 and *M. tuberculosis* H37Rv**

**Method**	**Running time (min′ s″)**
Proteinortho	05′ 03″
InParanoid	19′ 47″
OMA	91′ 48″
EGM2	0′ 18″
OrthoGNC (Conf 2 single round)	02′ 58″
OrthoGNC (Conf 8 iterative)	03′ 11″

*Note*: All methods were run on a personal computer with an Intel Core i7-4702MQ 2.20 GHz processor and 6 GB of RAM.

It is worth mentioning that OrthoGNC can also be used to find co-linear blocks within species. Simply, by adjusting parameters to *N* = *n*, *T*_n_ = *2n*, and NIR = “One2One Mapping”, the predicted orthologs will be centered in syntenic blocks of size 2*n* + 1. Finding syntenic blocks is of a great interest [42], [43], [44], because genes residing in a syntenic block have been under evolutionary pressure and are more likely to interact and be co-expressed [45].

## Conclusion

We have presented here OrthoGNC, a similarity-based software for detecting accurate orthology relations. To maintain higher accuracy, OrthoGNC is capable of inferring orthology relations in multiple rounds. OrthoGNC is very flexible and user-friendly in accepting user-defined parameters. Also, multithreaded implementation of OrthoGNC makes it fast and efficient for pipelines where high-quality orthology relations are needed. To achieve high specificity, OrthoGNC investigates genomic context of potential orthologs. Accuracy of OrthoGNC is validated by comparison against four competitive methods on both simulated and real data.

In addition to delivering accurate orthology relations, OrthoGNC can be employed to investigate the gene neighborhood conservation for refinement and assessment of other orthology inference methods.

## Authors’ contributions

CE and LW conceived the project and supervised the study. SJ, CE, and LW designed the study and evaluations. SJ developed the software and performed the evaluations. SJ, CE, and LW wrote the manuscript. All authors read and approved the final manuscript.

## Competing interests

The authors have declared no competing interests.
