# Supplementary figures and images for: OrthoGNC: A Software for Accurate Identification of Orthologs Based on Gene Neighborhood Conservation
Source: Genomics Proteomics Bioinformatics. 2017 Nov 11;15(6):361–70. doi: 10.1016/j.gpb.2017.07.002 (PMC5828658; doi:10.1016/j.gpb.2017.07.002)

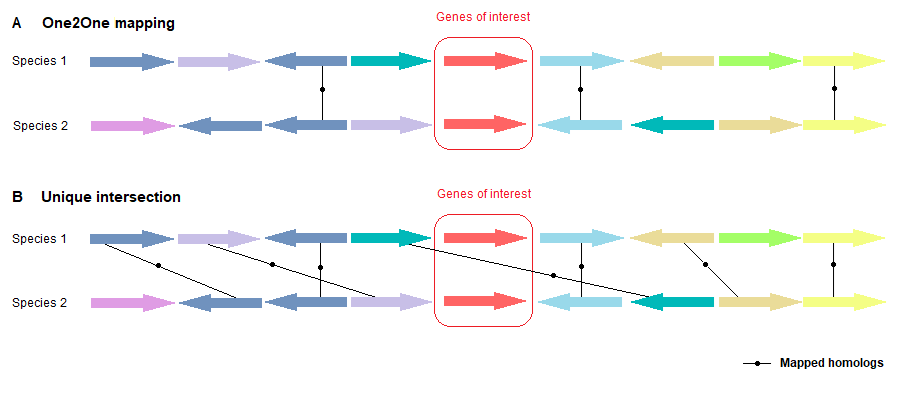

Supplement: Supplementary Figure S1 [file mmc1.zip › Figure S1 (Revised) ok.png]

## Slide 1
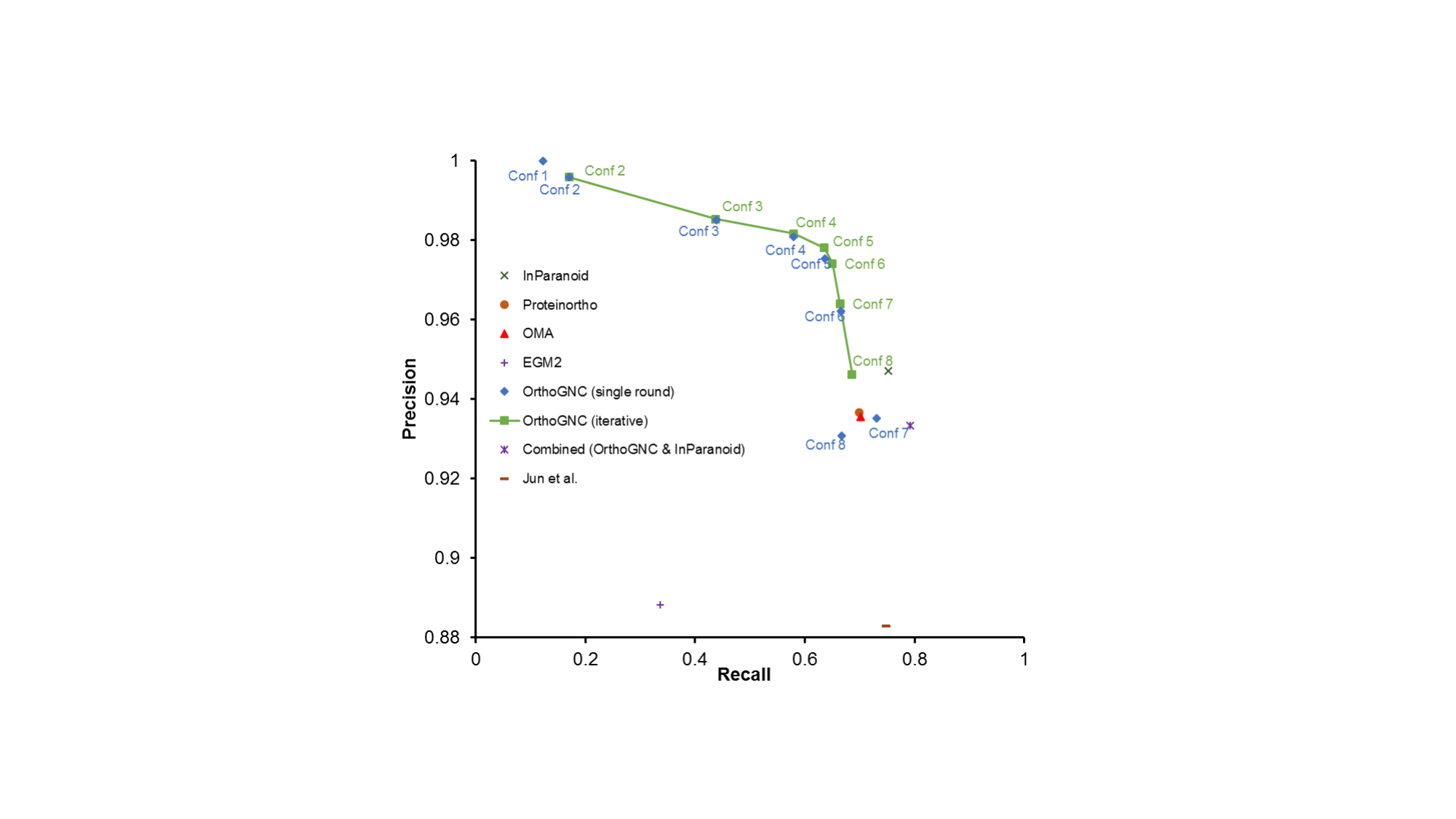

Supplement: Supplementary Figure S2 2 [file mmc2.pptx]

## Slide 1
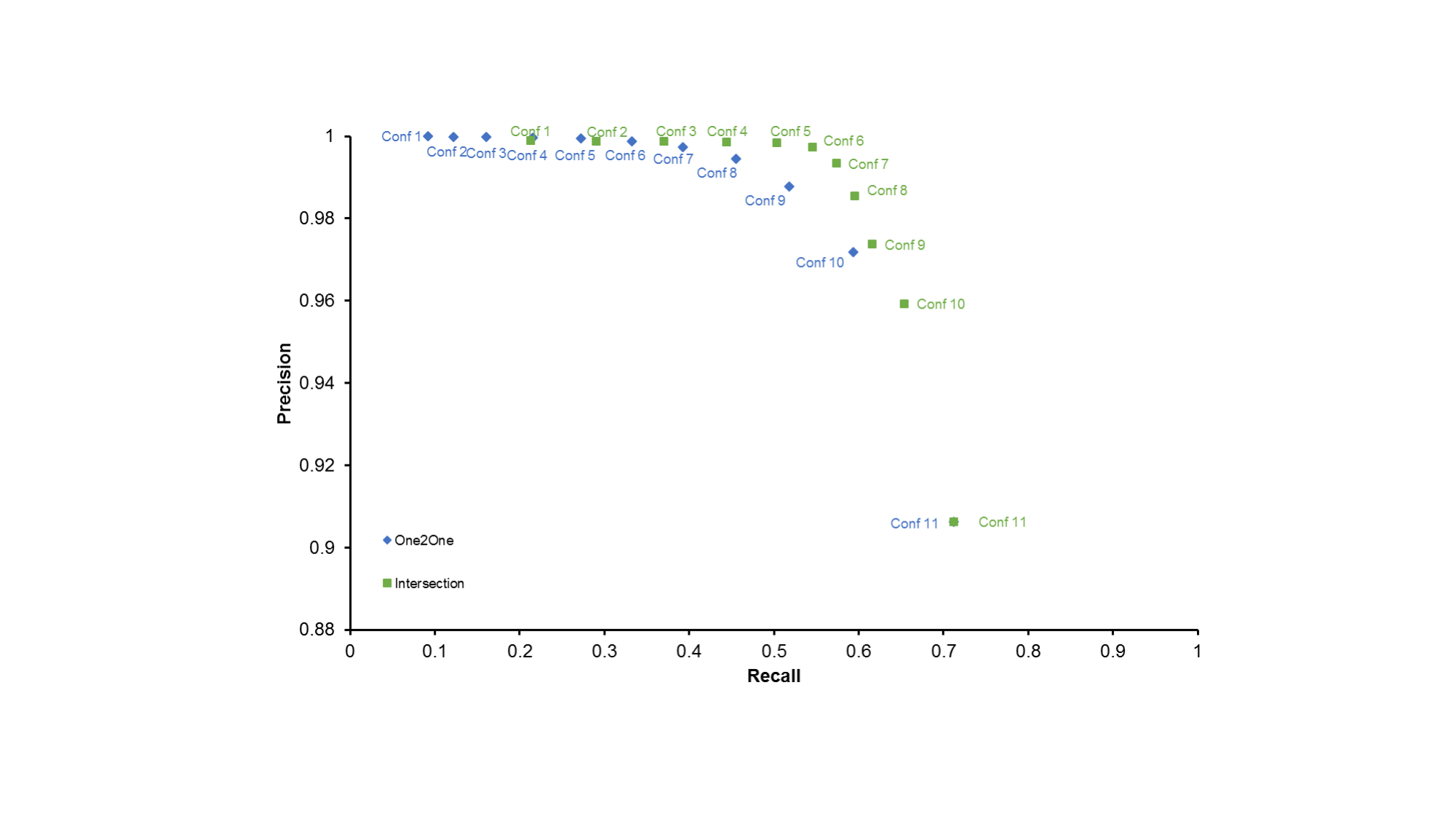

Supplement: Supplementary Figure S3 3 [file mmc3.pptx]
