# Supplementary material for: OrthoGNC: A Software for Accurate Identification of Orthologs Based on Gene Neighborhood Conservation
Source: Genomics Proteomics Bioinformatics. 2017 Nov 11;15(6):361–70. doi: 10.1016/j.gpb.2017.07.002 (PMC5828658; doi:10.1016/j.gpb.2017.07.002)
Supplement: Supplementary Table S1 [file mmc4.docx]

**Table S1 Parameters in 11 configurations used to generate Figure S3**

| Parameter | | Conf 1 | Conf 2 | Conf 3 | Conf 4 | Conf 5 | Conf 6 | Conf 7 | Conf 8 | Conf 9 | Conf 10 | Conf 11 |
| --- | --- | --- | --- | --- | --- | --- | --- | --- | --- | --- | --- | --- |
| Homology | E-value | 10^-5^ | 10^-5^ | 10^-5^ | 10^-5^ | 10^-5^ | 10^-5^ | 10^-5^ | 10^-5^ | 10^-5^ | 10^-5^ | 10^-5^ |
|  | T_i_ | 30% | 30% | 30% | 30% | 30% | 30% | 30% | 30% | 30% | 30% | 30% |
|  | T_c_ | 50% | 50% | 50% | 50% | 50% | 50% | 50% | 50% | 50% | 50% | 50% |
| Orthology | N | 7 | 7 | 7 | 7 | 7 | 7 | 7 | 7 | 7 | 7 | 7 |
|  | T_n_ | 10 | 9 | 8 | 7 | 6 | 5 | 4 | 3 | 2 | 1 | 0 |
|  | T_b_ | 0.80 | 0.80 | 0.80 | 0.80 | 0.80 | 0.80 | 0.80 | 0.80 | 0.80 | 0.80 | 0.80 |
|  | NIR | I/O | I/O | I/O | I/O | I/O | I/O | I/O | I/O | I/O | I/O | I/O |

*Note*: T_i_, minimum percentage of identical matches in a BLAST hit; T_c_, minimum percentage of coverage of query and subject sequences in BLAST hit; N, radius of neighborhood to be investigated; T_n_, minimum number of common neighbors (0 ≤ T_n_ ≤ 2*N); T_b_, maximum tolerance ratio from score of best hit (0 ≤ T_b_ ≤ 1); NIR, neighborhood investigation routine; I and O stand for unique intersection and One2One mapping, respectively.
